# Advances in Hyperspectral Imaging for Nondestructive Food Quality and Safety Detection

**DOI:** 10.3390/foods15101631

**Published:** 2026-05-07

**Authors:** Fayun Xing, Mingming Chen

**Affiliations:** Department of Microelectronics, Jiangsu University, Zhenjiang 212013, China; xingfayun@stmail.ujs.edu.cn

**Keywords:** hyperspectral imaging, nondestructive detection, food quality, food safety

## Abstract

Hyperspectral imaging (HSI) has become a reliable nondestructive method for evaluating food quality and safety, surpassing traditional methods that are typically destructive and labor-intensive. HSI integrates spectral signatures with spatial distribution, enabling real-time, high-sensitivity analysis of both internal and external food attributes. Recently, there has been a growing number of studies focusing on food quality and safety detection using the HSI technique. This review offers a comprehensive summary of advancements in detecting food quality and safety in key areas, such as assessing the quality of fruits, vegetables, meat, grains, and tea; measuring moisture content; identifying variety and geographic origin; detecting adulterants and additives; and evaluating heavy metals and pesticide residues. Additionally, challenges and perspectives, including data dimensionality, the trade-off between signal-to-noise ratio and resolution, hardware costs, and the gap between laboratory research and applications under variable environmental conditions, are proposed. This review highlights the great potential of the HSI technique for rapidly and nondestructively detecting and monitoring food quality and safety in food and agricultural applications.

## 1. Introduction

Since food is the foundation of human survival, food quality and safety profoundly influence human health, economic development, and social stability [1]. Generally, human health and lives are closely related to food safety. Typically, substandard foods may carry pathogenic microorganisms, chemical contaminants, and/or illegal additives, which lead to foodborne illnesses and further increase the risk of chronic diseases or even cancer [2,3,4]. Economically, with the development of modern agriculture and the food industry, strict food quality control is fundamental for sustainable development and for participating in international competition. However, food can be contaminated by biological agents (such as bacteria and parasites), chemical substances (such as pesticide residues, environmental pollutants, heavy metals, and illegal additives), and physical hazards at any stage of the supply chain, including production, processing, storage, transportation, and sales [5,6,7]. As a result, there is a growing need for efficient and nondestructive detection of food quality.

Traditional food quality sorting systems primarily depend on manual physical grading and color detection, which are generally laborious and time-consuming, and they cannot reveal internal features, such as damage, chemical substances, and contaminants. Various methods, including vibrational spectroscopy [8], enzyme-linked immunosorbent assay [9], mass spectroscopy [10,11,12], gas chromatography [11,12], thin-layer chromatography [13], liquid chromatography [14,15], and polymerase chain reaction [16,17,18], have been explored. Generally, these methods offer high sensitivity and specificity for the detection of pesticide residues, contaminants, and mycotoxins in various foods. However, they often destroy the samples, are labor-intensive and high-cost, and are not suitable for rapid and large-scale sample analysis. In addition, various spectroscopic analysis techniques, such as transmission [19,20], reflection [20], fluorescence [21], and Raman [22,23], have been studied for food quality inspection. Such technologies primarily rely on the spectral features of functional groups and molecules, such as C-H, C-N, and C=O, and pigment substances, such as chlorophyll, carotenoids, and anthocyanins, in food samples, showing great potential for nondestructive and effective food quality assessments. However, these single-point inspection techniques lack spatial analysis capabilities, which cannot detect variability in heterogeneous food samples [24].

In recent years, hyperspectral imaging (HSI) has gained remarkable interest in the food quality detection community owing to its unique ability to integrate imaging with spectral scanning [25,26]. It can provide detailed spectral and spatial data on samples in real time and efficiently identify internal characteristics with high sensitivity and accuracy. Very recently, HSI has gained widespread use in evaluating the internal and external quality of various foods (such as fruits, meat, vegetables, and grains) and crops (such as rice, wheat, and tea), aided by the regression models that link hyperspectral imaging data to specific target detection objects (such as chemical contaminants, moisture, and heavy metals) [27,28,29]. Currently, this technology has emerged as a powerful and nondestructive method for rapidly evaluating food quality to guarantee food security.

**Figure 1:**
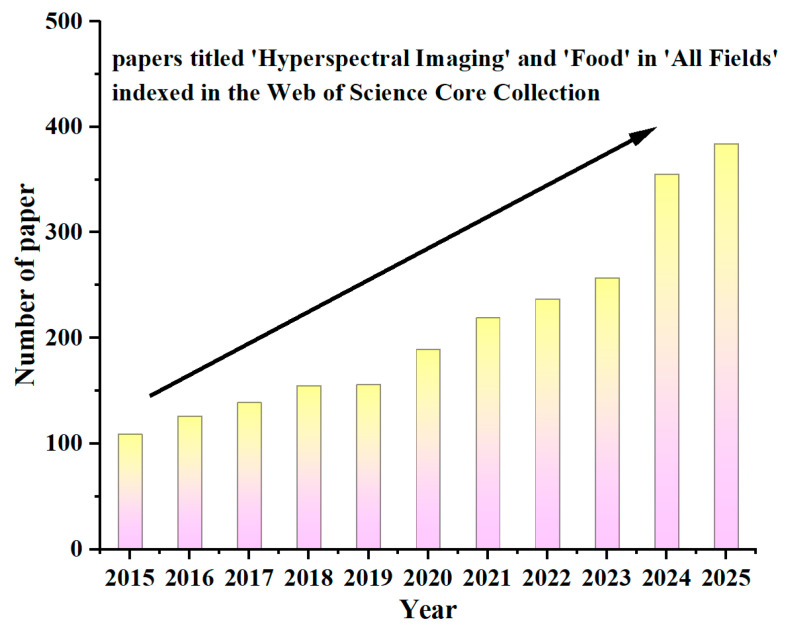
Number of papers titled ‘Hyperspectral Imaging’ and ‘Food’ in ‘All Fields’ indexed in the Web of Science Core Collection in the past ten years.

In recent years, the number of published papers has increased steadily (Figure 1), showing that HSI technology remains a prominent research area. Meanwhile, several reviews have been reported to summarize the typical achievements in the HSI technique for detecting food quality, identifying plant diseases and pests, and monitoring crop growth [30,31,32,33,34,35,36,37], which typically focus on a single food category (e.g., cereals [34], corn [32]), a singular methodological emphasis (such as deep learning [35]), or a narrow application scenario (disease detection [33,37], foreign object detection [30]). This review offers a comprehensive overview of HSI technology integrated with machine learning and deep learning techniques, emphasizing its recent applications in assessing the quality and safety detection of various types of food. It is organized as follows: Section 2 elaborates on the basic principles and system components of HSI, as well as hyperspectral image data processing. Section 3 presents typical advances in detecting food quality and safety using HSI technology. Typical applications, such as quality assessment, moisture content detection, variety and origin identification, and detection of additives, heavy metals, and pesticide residues, are summarized. Finally, the review addresses current challenges and prospects for future research and technological progress.

## 2. Basics of Hyperspectral Imaging Technology

### 2.1. Principles of Hyperspectral Imaging

**Figure 2:**
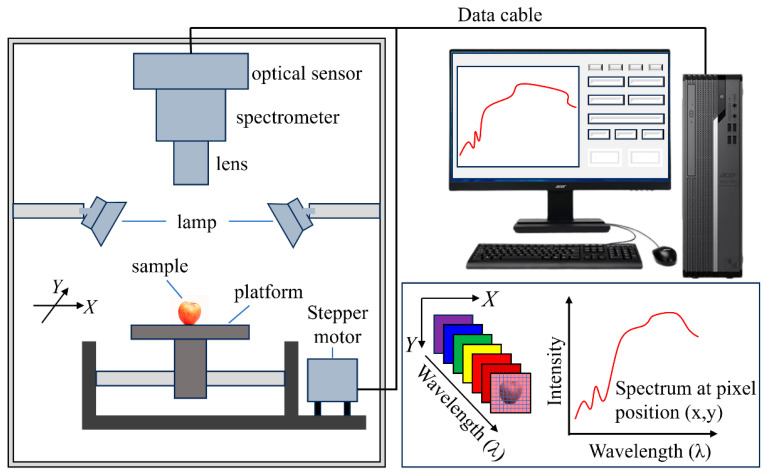
Schematic diagram of the HSI system. The inset shows the three-dimensional (3D) data cubes consisting of two-dimensional spatial (*x*, *y*) data and one-dimensional spectral (*λ*) data.

HSI is a highly integrated system that combines optics, mechanics, electronics, and information processing. As shown in Figure 2, the system includes optical imaging and spectroscopic units, image sensors, sample platforms, illumination units, data acquisition and control systems, and data processing and analysis software. Specifically, the optical imaging and spectroscopic units mainly consist of lenses and spectroscopic devices, such as prism gratings or tunable filters, which analyze the spectral information transmitted or reflected from targets in the spatial domain. The image sensors, typically high-sensitivity silicon-based CCD and CMOS arrays, capture both spatial images and spectral data for each pixel across numerous narrow spectral bands. The illumination units provide stable and controllable light sources (such as halogen lamps or LED arrays) with wavelengths spanning the 300–2500 nm spectral range and are usually equipped with white reference panels to ensure spectral data accuracy and repeatability. The data acquisition and control systems include high-speed data interfaces, synchronization controllers, and motion modules that coordinate spectral collection, spatial scanning, and signal transmission. The software for data processing and analysis provides functions such as radiometric calibration, spectral correction, feature extraction, chemometric modeling, and visualization, transforming the raw data into spectral images suitable for qualitative and quantitative analysis. The entire system integrates physical signal collection with digital spectral image generation through its various interconnected components [38]. Notably, to obtain accurate information from small samples and from microscope measurements, microscopy can be integrated into the optical path [39,40].

**Figure 3:**
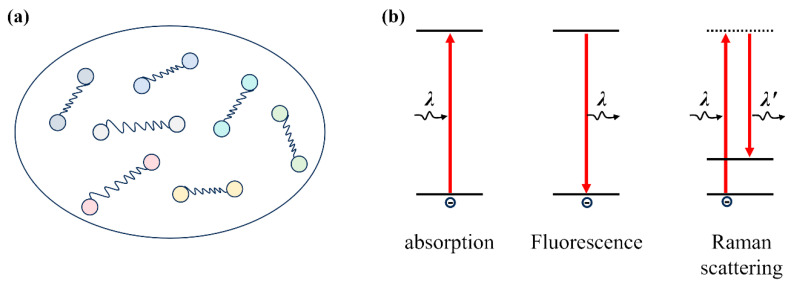
(**a**) Scheme of molecular vibrations (shown as strings) in foods. (**b**) Typical photo-electron interaction processes of absorption, fluorescence, and Raman scattering.

As shown above, HSI combines optical imaging with spectroscopy. The spectroscopy component provides the foundation for evaluating the internal features of food samples, which are mainly related to interactions between photons and matter. The basic principles can be found in the Semiconductor Optics textbook [41]. As organics, foods are composed of various kinds of organic molecules and functional groups (such as H-O, C-N, C=C, and C=O). As shown by quantum mechanics, molecules and functional groups vibrate at specific frequencies (i.e., molecular vibrations, represented by the various strings in Figure 3a). Notably, these vibrational frequencies differ among molecules and functional groups. When light illuminates food samples, photons interact with molecular vibrations, creating characteristic peaks (Figure 3b) in reflection, absorption, and transmission spectra, usually within the near-infrared (NIR) and mid-infrared (MIR) ranges. More specifically, photons with specific energies are absorbed by these molecules and groups, while the unabsorbed photons are reflected or transmitted. Accordingly, the transmission and reflection spectra contain various information, including internal state, chemical composition, and external quality of food samples. In other words, the detailed information of food samples is encoded in the HSI data. This scenario serves as the basis for IR and Raman spectroscopy, which have been widely employed to analyze molecules and chemical bonds in materials science [42].

Generally, different organic compounds are composed of different functional groups. Typically, amino acids contain amino (-NH_2_) groups, which exhibit characteristic peaks in the 3300–3500 cm^−1^ range [43]. Carbohydrates contain various hydroxyl (-OH) and ether oxygen groups (C-O-C), with characteristic peaks in the 3600–3100 cm^−1^ and 950–1300 cm^−1^ ranges, respectively [44,45]. In contrast, carboxylate compounds contain carbonyl (C=O) groups, which have characteristic peaks around 1700 cm^−1^ [45,46]. Proteins and polypeptides contain various acylamino (-CO-NH-) groups, with characteristic peaks around 3300 cm^−1^, 3060 cm^−1^, 1650 cm^−1^, and 1530 cm^−1^ [47,48,49]. In practice, the quality of vegetables and fruits has been studied by measuring pigment compounds such as chlorophyll, carotenoids, and anthocyanins [50,51,52,53,54,55], which show characteristic peaks in the visible spectrum. For example, the characteristic peaks of chlorophyll appear at around 430 nm and 660 nm in the absorption spectrum, and around 550 nm in the transmission and reflection spectra. In comparison, carotenoids show characteristic peaks at around 450 nm and 480 nm in the absorption spectrum, and in the 500–700 nm spectral range in the transmission and reflection spectra. Generally, the amount of chlorophyll in vegetables and fruits shows their freshness and degree of ripeness [51,55,56]. The content of carotenoids reflects the ripeness and quality of fruits [54,57]. The characteristic wavelengths and key roles of typical pigment compounds are shown in Table 1. In theory, the characteristic peaks of bulk materials are narrow. However, since pigment compounds are randomly dispersed in foods and doped with various elements, the characteristic peaks tend to be broad and often overlap.

**Figure 4:**
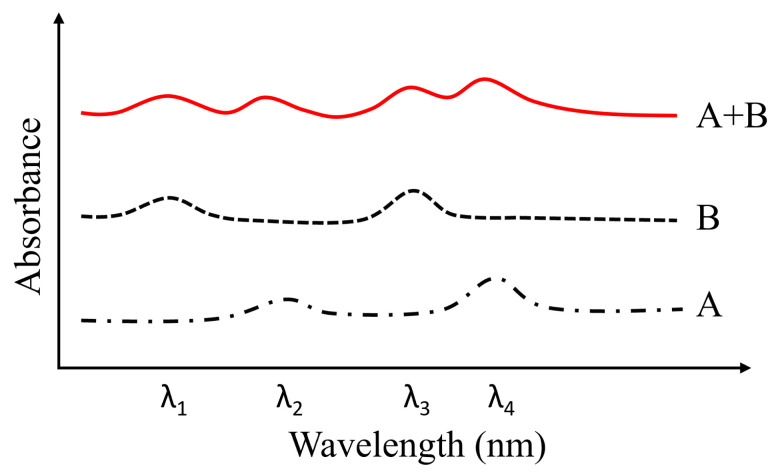
Schematic diagram of the absorbance spectra of food samples consisting of compounds A (black dash–dot line), B (black dashed line), and A + B (red line).

Apart from revealing the chemical composition of the food samples, their amounts are also important. As mentioned above, the amount of chlorophyll in vegetables indicates their freshness [55]. More importantly, the pesticide residue levels in fruits and vegetables affect human health. Interestingly, the amount (content) of various chemical compositions and substances can be quantitatively assessed according to the Lambert–Beer law: $I_{T}=I_{in}e^{-\alpha d\rho }$, where *I*_T_ and *I*_in_ are the intensities of transmitted and incident light, respectively, α is the molar absorption coefficient (L·mol^−1^·cm^−1^), *d* is the thickness of food samples, and ρ is the concentration (mol/L) of the target detection objects. Here, *αdρ* is the absorbance of the target detection objects. Accordingly, the transmittance (*T*, $T=I_{T}/I_{in}$) shows the absorbance, based on which the amounts of chemical compositions and substances can be obtained. Ideally, absorbance exhibits superposition. For example, when light illuminates a food sample that is made of compound A and compound B, the overall absorbance equals the sum of the absorbance of compound A and that of compound B (Figure 4). Such a phenomenon increases the complexity of HSI measurements, as food samples generally contain a wide range of chemical compositions and functional groups. Notably, for turbid foods, absorbance measurements of chemical composition and substances from transmittance may be inaccurate owing to high scattering, but these can be corrected using the Kubelka–Munk principles. To accurately estimate the amounts of each component, the HSI data should be carefully preprocessed and analyzed, and regression models should be further constructed, typically using well-established deep learning and machine learning methods [26,33,35,58], as shown below. However, since CCD detection has limited sensitivity to light intensity, the detection limits for chemical compositions and substances in foods are typically at the mg/kg and mg/L levels.

### 2.2. Hyperspectral Image Acquisition

**Figure 5:**
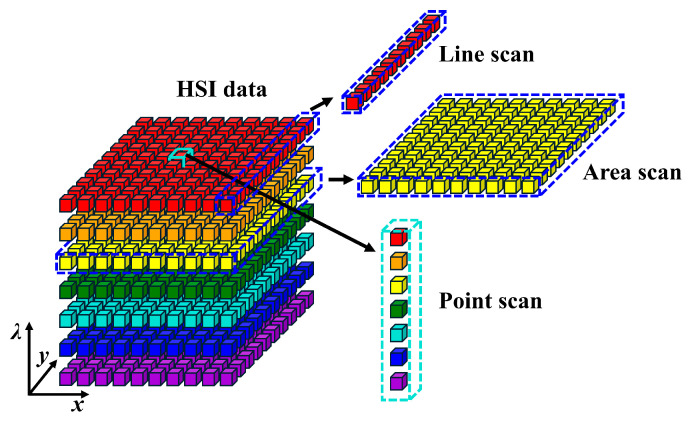
Typical modes for acquiring HSI data.

In practice, HSI measurements can be conducted in different modes such as reflection, transmission, and scattering (Figure 5) [34]. In reflection mode, the camera and light source are on the same side, and the camera captures reflected light from the sample surface. In this mode, both the IR and visible spectral signals can be recorded but mainly originate from the surface of food samples. It primarily assesses the external features of the samples, such as size, color, and surface defects. In comparison, in transmission mode, the camera and the light source are located on opposite sides of the food samples, where the camera primarily records the transmitted light from the food samples. In this mode, IR spectral signals are generally captured, which can be decoded to reveal the internal features, such as compositions and additives. However, due to the relatively low intensity of scattered light, the scattering mode is less commonly used in practice.

As mentioned above, HSI technology integrates detailed spatial and spectral data of samples, demonstrating that hyperspectral images are three-dimensional (3D) data cubes consisting of two-dimensional spatial (*x*, *y*) data and one-dimensional spectral (*λ*) data (Figure 2 inset). The former is generally defined by the coordinates of pixels of cameras, and the latter contains the spectral information of food samples. Hyperspectral images can be recorded using point, line, or area scanning (Figure 5). In point-scanning mode, either the camera or the food sample moves along the *x*- or *y*-axis. It captures the full spectral data of a single pixel at a time and then combines them to form the hyperspectral images. In practice, point scanning yields high accuracy but is time-consuming. In line-scanning mode, either the camera or the food sample moves along a predetermined path to collect spectral data for each pixel in a line. In the area-scanning mode, the entire sample image is sequentially captured at each wavelength, with repeated scans across the full spectrum. Compared to point- and line-scanning modes, the area-scanning mode is efficient for applications that require data from multiple wavelengths.

### 2.3. Hyperspectral Image Data Processing

**Figure 6:**
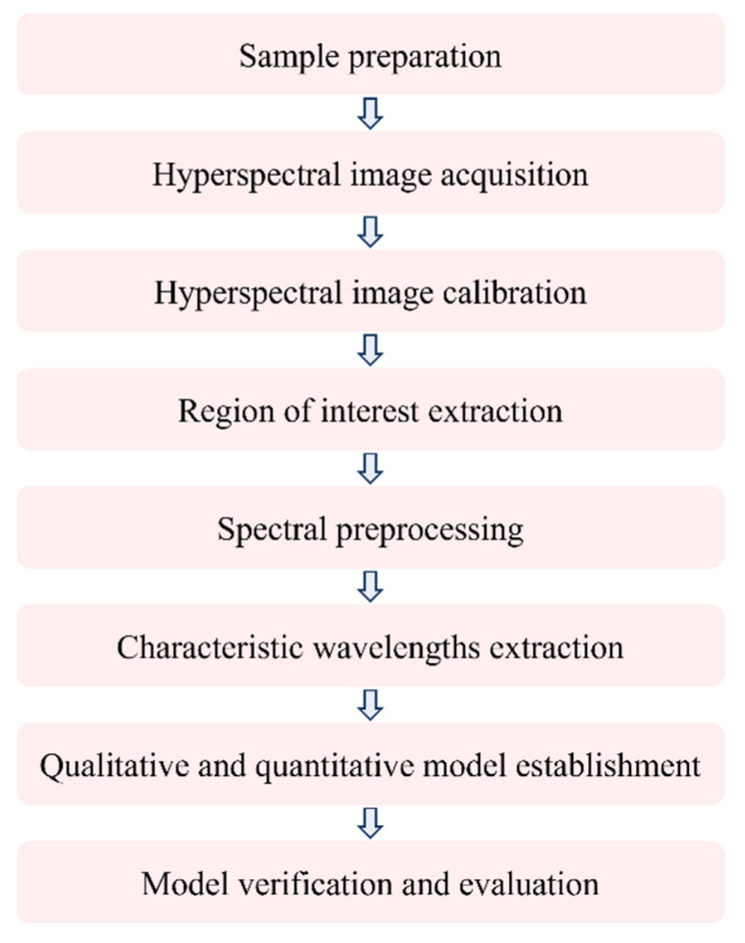
Typical workflow for processing hyperspectral imaging data.

In practice, detailed information, such as chemical compositions and contents, is deeply concealed within the hyperspectral images [59]. Following the acquisition of hyperspectral images, proper data processing is essential for revealing the chemical compositions and contents within the food samples. Generally, four challenges limit the accuracy of HSI results. First, the captured hyperspectral images contain various types of noise, including electrical noise from the HSI system and environmental noise. Second, additional factors, such as the volume and surface of food samples, also affect the intensity of hyperspectral images, along with their chemical composition and content. As a result, the composition and contaminant content cannot be directly inferred from hyperspectral image intensities. Third, characteristic peaks of chemical compositions and contaminants usually overlap in spectral data [60]. In this situation, it is challenging to precisely identify the characteristic wavelengths in the hyperspectral images. Finally, for reasons similar to those in the third challenge, the content of detection objects cannot be directly inferred from the intensities of their characteristic peaks. Experimentally, three main stages of data processing, such as image noise reduction and spectral correction (known as *preprocessing*), characteristic wavelength extraction, and predictive model construction, should be carefully performed to accurately visualize the results of chemical compositions and contents within the food samples. The first stage enhances the signal-to-noise ratio of the raw hyperspectral image data, the second stage extracts characteristic wavelengths of target detection objects, and the third stage provides comprehensive data on chemical composition and content. Figure 6 illustrates the typical workflow for processing hyperspectral imaging data.

Thanks to significant advances in signal processing, deep learning (DL), and machine learning (ML) over the past years [61,62,63], the above-mentioned issues have been effectively addressed. Typically, the quality of dried wolfberry fruit has been assessed with an accuracy of 96.66%, achieved by using standard normalization variate (SNV) and Savitsky–Golay (SG) methods to improve hyperspectral image quality by cleaning data and eliminating particle-size interferences [64]. Partial least squares (PLS) regression models were developed to predict the chemical composition of cheeses, including moisture, protein, and fat content [65,66]. In another study, a modified supervised locality preserving projections (MSLPP) based ML method was used to extract characteristic wavelengths that retained global information and local structure of moisture in rice, achieving 97.55% accuracy in detecting the moisture content [67]. In addition, Zhang et al. have reported the effective detection of levels of capsaicin and hydroxy-α-sanshool in spicy foods during the hotpot seasoning process with the HSI technique combined with the min-max scaler method to preprocess the hyperspectral imaging data [68]. Table 2 shows the methods for hyperspectral image preprocessing, characteristic wavelength extraction, and predictive model construction reported in recent years, where the critical roles of typical algorithms are summarized in refs. [26,30].

## 3. Advances in Hyperspectral Imaging for Food Quality and Safety Detection

### 3.1. Hyperspectral Imaging for Quality Assessment

#### 3.1.1. Fruit and Vegetable Quality

**Figure 7:**
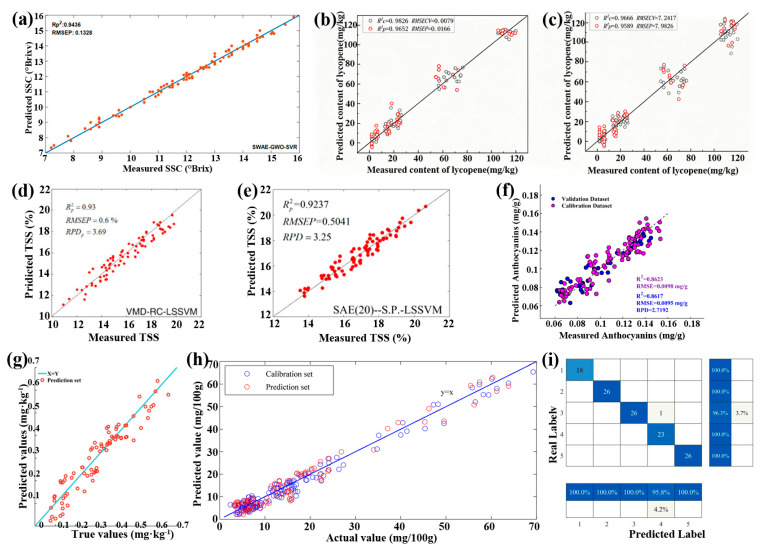
Measured and predicted (**a**) soluble solid content in apples by the SWAE-GWO-SVR model. Reproduced with permission from Ref. [71]; (**b**,**c**) lycopene content in tomatoes by (**b**) CARS-SVR and (**c**) CARS-PLSR models [69]; (**d**,**e**) total soluble solid content in grapes by (**d**) VMD-RC-LSSVM (reproduced with permission from Ref. [92]) and (**e**) SAE-LSSVM (reproduced with permission from Ref. [93]) models; (**f**) anthocyanins content in lettuce by the UVE-SNV-CARS-DBO-ELM model [70]; (**g**) selenium content in lettuce by MDCARS-RCNN (reproduced with permission from Ref. [99]); (**h**) bud eye in potatoes by the SVR model (reproduced from with permission from Ref. [97]); and (**i**) confusion matrix of the POA-CDGSA-Net model for assessing kiwifruit quality (reproduced with permission from Ref. [100]).

Fruits and vegetables provide essential vitamins, minerals, and dietary fiber necessary for a healthy immune system. Their quality, however, depends on cultivation and storage methods [89,90]. Recently, the HSI technique has been studied for the nondestructive detection of the quality of various fruits and vegetables, such as tomatoes [69,91], grapes [92,93,94], apples [71,95], cape gooseberry [96], potatoes [97,98], lettuce [70,99], and kiwifruits [100,101]. In practice, the quality of fruits and vegetables is assessed based on the content of various specific components, which are detected using the HSI technique combined with DL and ML (Table 2). As mentioned earlier, the latter are used to extract characteristic wavelengths and further construct prediction models. Typically, Tian et al. have employed the HSI technique to assess the quality of apples nondestructively and rapidly by measuring their soluble solid content (Figure 7a), where the characteristic wavelengths were extracted from the SWAE DL technique [71]. In addition, Saavedra et al. have detected the vitamin C content, firmness, soluble solids content, and titratable acidity in cape gooseberry using the near-infrared HSI technique [96]. In another report, Dai et al. assessed the maturity of tomatoes according to their lycopene content, which was obtained by the HSI technique combined with the CARS algorithm to extract characteristic wavelengths and SVR and PLSR prediction models (Figure 7b,c) [69]. Similarly, Taha et al. monitored the growth stage of aquaponically grown lettuce by detecting the chlorophyll content using the HSI technique combined with an open-source automated machine learning algorithm [102]. It is worth noting that the HSI technique is highly accurate for assessing the quality of fruits and vegetables. For example, the coefficient of determination (*R*^2^) and the root mean square error for the prediction (RMSEP) of soluble solid content were obtained as high as 0.944 and 0.133 °Brix in Ref. [71]. In addition, the maximized *R*^2^ and RMSEP in detecting lycopene content are approximately 0.965 and 0.017 mg/kg in Ref. [69]. Notably, high accuracy has been further achieved in assessing grape quality, which has been realized by measuring their soluble solids and titratable acidity contents using the HSI technique combined with the VMD-RC-LSSVM [92] and the SAE-LSSVM [93] algorithms, with the *R*_P_^2^ values of 0.93 and 0.92, respectively (Figure 7d,e). Significantly, the model’s accuracy can be further enhanced by using explainable artificial intelligence to interpret the prediction models and assess the contribution of the variable wavelengths [103,104]. In addition, the nutritional content of vegetables has also been assessed using the HSI technique. Typically, the anthocyanin and selenium content, which determine the nutritional value of lettuce, have been rapidly and accurately detected using the HSI technique either combined with UVE-CARS-SNV-DBO-ELM [70] or MDCARS-RCNN [99] mixed algorithms, respectively (Figure 7f,g).

Furthermore, the HSI technique has also been explored to detect by-products and harmful materials in various fruits and vegetables produced during storage. For example, Lu et al. have shown the nondestructive and rapid detection of solanine content in potatoes, which causes nerve center paralysis and other discomfort symptoms [105], using the HSI technique. The characteristic wavelengths were extracted using a single CARS algorithm, based on which an optimized SVR model was constructed to predict the solanine content, with *R*^2^ and RMSEP of 0.9143 and 0.0296, respectively (Figure 7h) [97]. Very recently, Cong et al. have reported the effective and rapid detection of early chilling injury in kiwifruits using the HSI technique combined with the POA-CDGSA-Net hybrid model (Figure 7i) [100]. Table 3 shows the typical results of recent quality assessments of fruits and vegetables using the HSI technique.

#### 3.1.2. Meat Quality

**Figure 8:**
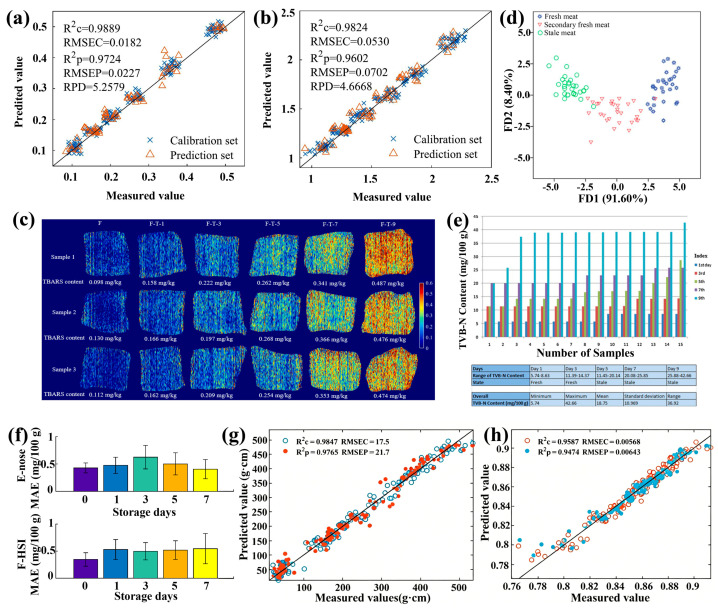
Measured and predicted (**a**) TABRS and (**b**) carbonyl content in pork samples by a multi-task CNN model. Reproduced with permission from Ref. [113]. (**c**) Visualization of lipid oxidation degree of pork samples. Reproduced with permission from Ref. [115]. (**d**) Score scatter plot with two Fisher discriminant analysis factors of three groups of pork samples [116]. (**e**) Bar chart and statistics table of chemical reference measurements of chicken breast fillets. Reproduced with permission from Ref. [117]. (**f**) Predictive performance of hybrid fusion attention network for the assessment of pork freshness. Reproduced with permission from Ref. [118]. Measured and predicted (**g**) gel strength and (**h**) water-holding capacity in surimi during their two-stage water bath heating processes. Reproduced with permission from Ref. [124].

Meat, such as chicken, pork, and beef, provides essential proteins, micronutrients, and fats for humans [112]. Before consumption, it undergoes pre-treatment and processing steps, typically freezing and drying, during which pathogen growth and lipid oxidation occur simultaneously. The HSI technique has been shown to be effective in nondestructively and rapidly detecting meat quality. Recently, Cheng et al. reported the detection of lipid oxidation and protein oxidation in pork using the HSI technique in combination with various algorithms and models, including the MI-VIF algorithm [83], multi-task CNN [113], lightweight 3D-CNN [114], and GPR model [115]. In practice, the algorithms and models used determine the accuracy of predictions, since the HSI data contains numerous pieces of information as shown in Section 2.3. Specifically, the PLSR prediction models based on characteristic wavelengths extracted from the MI-VIF algorithm predicted carbonyl content with an R_p_^2^ of 0.9275 and an RMSEP of 0.0812 nmol/mg, and sulfhydryl content with an R_p_^2^ of 0.9512 and an RMSEP of 1.2979 nmol/mg [83]. The multi-task CNN model predicted lipid oxidation and protein oxidation with R_p_^2^ values of 0.9724 and 0.9602, and RMSEPs of 0.0227 and 0.0702, respectively (Figure 8a,b) [113]. The lightweight 3D-CNN model combined with the 2D-COS analysis predicted thiobarbituric acid-reactive substances (TBARS) with an R_p_^2^ of 0.924 and an RMSEP of 0.0364 mg/kg [114]. Interestingly, the accuracy of the GPR model in predicting TBARS has improved, with an R_p_^2^ of 0.9726 and an RMSEP of 0.0182 mg/kg, based on which the lipid oxidation degree of pork was visualized (Figure 8c) [115]. Additionally, the HSI technique combined with the BPANN model has been further explored for assessing the freshness of pork [116] and chicken [117] by detecting volatile basic nitrogen content, achieving 100% accuracy in the former case and an RMSEP of 6.3834 mg/100 g in the latter case (Figure 8d,e). Recently, Cheng et al. demonstrated a novel hybrid fusion attention network that incorporates early fusion with an attention mechanism into late fusion to improve the accuracy of the assessment of pork freshness (Figure 8f) [118]. Notably, additional testing techniques, such as colorimetric sensor array-based artificial olfaction [119], gas chromatography-ion mobility spectrometry (IMS), and confocal imaging [120,121], have also been combined with the HSI technique to detect moisture content, total viable count, and volatile components for the nondestructive and rapid assessment of pork quality. It is worth noting that additional preprocessing of raw HSI data from packaged meats is required, since the packaging material significantly affects the spectral reflectance of samples, which greatly reduces model accuracy. Wu et al. reported the detection of lipid oxidation content in raw beef, with an R_C_^2^ of 0.9257 for the unpackaged samples, and R_C_^2^s of 0.7858 and 0.8798 for the packaged samples without and with Gaussian filter preprocessing [122]. To sum up, Table 4 presents the typical results for meat quality assessment using the HSI techniques.

**Table 4:** Typical results of recent meat quality assessment using the HSI technique.

Objective	Accuracy for Training Set		Accuracy for Test Set		Ref.
	*R* ^2^	RMSEC	*R* ^2^	RMSEP	
TVB-N in chicken	0.9821	2.2794 mg/100 g	0.7542	6.3834 mg/100 g	[117]
TVB-N in shrimp	0.9770	1.58 mg/100 g	0.9431	2.49 mg/100 g	[82]
Lipid oxidation in shrimp	0.9943	1.21%	0.9815	2.17%	[82]
TVB-N in pork	0.9616	0.4826 mg/100 g	0.9373	0.4897 mg/100 g	[118]
TVB-N in lamb	0.9131	2.9527 mg/100 g	0.9006	3.0742 mg/100 g	[123]
Carbonyl in pork	0.9305	0.1011 nmol/mg	0.9257	0.0812 nmol/mg	[83]
Sulfhydryl in pork	0.9550	1.6096 nmol/mg	0.9512	1.2979 nmol/mg	[83]
TBARS in pork	0.9341	0.0340 mg/kg	0.9214	0.0364 mg/kg	[114]
Gel quality of surimi	0.9426	0.6595	0.9363	0.7168	[124]
TBC in pork	0.9165	2.819 lg(CFU/g)	0.9055	2.991 lg(CFU/g)	[119]
TBC in lamb	0.94	0.76 lg(CFU/g)	0.91	0.84 lg(CFU/g)	[125]
Deterioration of beef	0.8798	0.1951 mg/kg	0.8309	0.2189 mg/kg	[122]
Pseudomonas in beef	0.9415	0.70 lg(CFU/g)	0.8636	1.05 lg(CFU/g)	[126]
Lactobacillus in beef	0.7381	0.58 lg(CFU/g)	0.7101	0.79 lg(CFU/g)	[126]

**Note:** TVB-N (total volatile basic nitrogen) is an indicator of the degree of chicken spoilage. TBARS (thiobarbituric acid reactive substance) is an indicator of the degree of lipid oxidation. Total bacterial count (TBC) is an important microbiological parameter for the sanitary and safety evaluation of meat. The reported performance metrics are study-specific and should be interpreted with caution.

Furthermore, the HSI technique has proven to be effective for monitoring the quality of various meats during processing. Recently, Xia et al. reported that the HSI technique combined with either PLS or CNN-LSTM models exhibited promise for monitoring the physicochemical properties and gel quality of surimi products during two-stage water-bath heating processes (Figure 8g,h) [124]. Aheto et al. studied the crystal-size effect on sodium chloride uptake and water activity of dry-cured pork using the HSI technique [127]. Li et al. reported the effective detection of physical quality attributes, including hardness and elasticity, of chilled lamb using the HSI technique combined with a dimensionality-reduction PSLR model [128]. In addition, the HSI technique has been used to assess the quality and condiment distribution in processed beef, such as marinated beef after Gaidao processing [129] and cooked beef [130]. Meanwhile, the HSI technique, combined with the ML and DL methods, has been further employed to visualize various chemical components in shrimp flesh, such as total volatile basic nitrogen and lipid oxidation [35].

#### 3.1.3. Grain Quality

**Figure 9:**
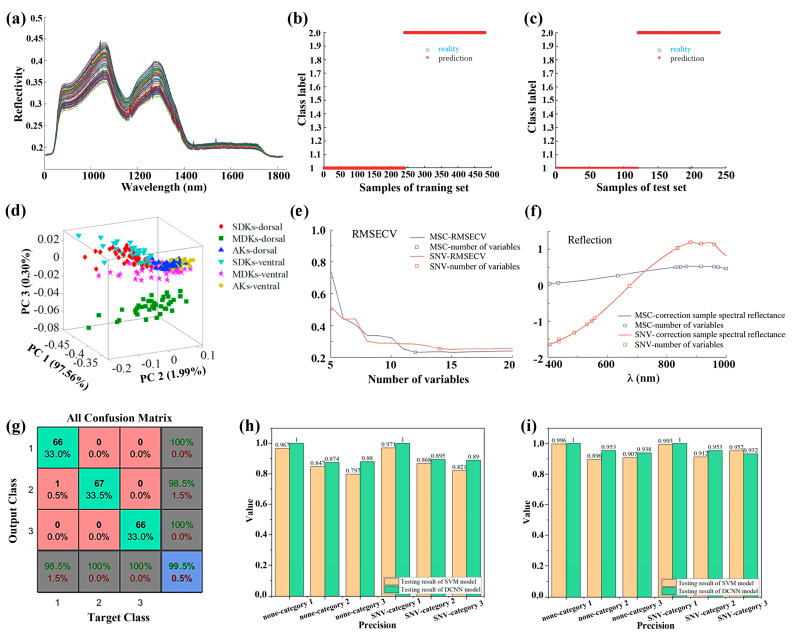
(**a**) As-captured spectra of rice samples. Reproduced with permission from Ref. [132]. (**b**,**c**) Identification results of the CARS-SPA-SVM model for the detection of selenium content in millet for (**b**) the training and (**c**) the test sets [133]. (**d**) PCA scores of dorsal and ventral spectra of severely damaged kernels, moderately damaged kernels, and asymptomatic kernels. Reproduced with permission from Ref. [136]. (**e**) RMSECV and (**f**) characteristic wavelengths identified through wavelength selection by the SPA method. Reproduced with permission from Ref. [137]. (**g**) Confusion matrices for the detection of mites in flour with the ACO-PCA-ANN mixed algorithms. Reproduced with permission from Ref. [138]. (**h**,**i**) Assessment accuracies of the test set for assessing corn seed freezing damage based on hyperspectral data from (**h**) the endosperm side and (**i**) the embryo side [140].

Grains, such as rice, corn, and wheat, are the primary sources of energy and nutrients for humans, offering essential carbohydrates and dietary fiber to humans worldwide. The quality of grains and their safety, considering their nutritional content and mold presence, impact human health and are vital for avoiding systemic health issues [131]. Nondestructive and rapid detection of grain quality and safety has high research significance. Recently, the HSI technique combined with various algorithms and models has been widely explored to nondestructively and rapidly detect the nutrients (including starch and micronutrients) and pathogens in various grains. Typically, Lu et al. reported quantitative detection of rice starch using the HSI technique (Figure 9a) [132]. In their work, the PCA algorithm was employed to extract characteristic wavelengths, and the SVR model was constructed to detect the starch content, achieving an R_p_^2^ of as high as 0.991 and an RMSEP of 0.669%. Zhang et al. reported rapid and nondestructive identification of selenium content in millet using the HSI technique combined with CARS-SPA mixed algorithms to extract the characteristic wavelengths and an SVM model to assess the selenium content, with an accuracy of 100% in the training set and 99.58% in the test set (Figure 9b,c) [133].

Apart from assessing nutrients, detecting toxins in grains is critical. Generally, grains tend to develop mold colonies during storage, which produce deoxynivalenol that causes emesis, elicits anorexia, and further impairs growth and production [134]. Erkinbaev et al. demonstrated that wheat damage from *fusarium* and *ergot* can be effectively detected simultaneously using the HSI technique combined with the PLSDA model, achieving an accuracy of over 90% [135]. Recently, Shen et al. have reported the rapid and nondestructive detection of deoxynivalenol in wheat kernels using the HSI technique [136]. Local PLS based on global PLS scores algorithms was employed for building quantification models of deoxynivalenol with an R_p_^2^ of 0.81 and an RMSEP of 40.25 mg/kg (Figure 9d). In another report, an improved classification accuracy of 100% for the training set and 97.92% for the testing set in assessing the deoxynivalenol content was obtained in an SVM model built based on characteristic wavelengths extracted from the single SPA algorithm (Figure 9e,f) [137]. In addition, the HSI technique has also been explored for the rapid and accurate detection of *Tyrophagus putrescentiae* and *Cheyletus eruditus* in wheat flour, achieved by combining with the ACO-PCA-ANN mixed algorithms with an accuracy of 98% (Figure 9g) [138]. Recently, Yang et al. reported identifying multiple Aspergillus flavus strains growing within peanut kernels using the line-scan Raman HSI technique combined with CARS-SVM mixed algorithms, effectively identifying three Aspergillus strains, namely A. flavus 142801, 142803, and 336156 [139].

In addition to evaluating nutrient and toxin levels, the HSI technique has been further explored for detecting freezing damage in various seeds. Very recently, Zhang et al. reported an effective assessment of freezing damage of core seeds using the HSI technique combined with a deep CNN method, showing an accuracy higher than 94% (Figure 9h,i) [140]. Notably, a similar accuracy has been demonstrated with the linear discriminant analysis (LDA) model constructed using characteristic wavelengths extracted from SPA-2D correlation analysis (2DCOS) mixed algorithms [141].

#### 3.1.4. Tea Quality

**Figure 10:**
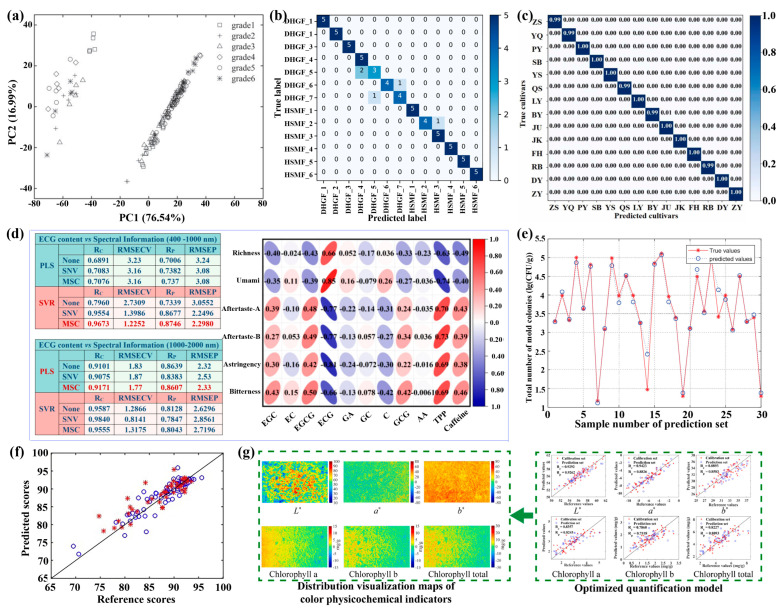
(**a**) Cluster plot of preliminary PCA model for assessing the Huangshan Maofeng tea quality. Reproduced with permission from Ref. [143]. (**b**) Confusion matrix for assessing the Huangshan Maofeng tea quality of the TSPSO-ResNet-50 model. Reproduced with permission from Ref. [144]. (**c**) Confusion matrix for evaluating cross-cultivar tea classification with the 1D-ResNet18 model. Reproduced with permission from Ref. [145]. (**d**) Prediction of epicatechin gallate (ECG) from the HSI technique, serving as an indicator to assess the appearance and taste quality of dry tea. Reproduced with permission from Ref. [146]. (**e**) Prediction of the total mold colony count in green tea based on MSC-CARS-GA-PSO-SVR algorithms. Reproduced with permission from Ref. [147]. (**f**) Comparison between predicted sensory scores and reference scores in the calibration set (marked with circle) and prediction set (marked with asterisk) for overall sensory attributes in CARS-ANN models. Reproduced with permission from Ref. [148]. (**g**) Predicting and visualizing matcha color physicochemical indicators using the HSI technique combined with chemometrics and various algorithms. Reproduced with permission from Ref. [149].

Tea drinks have been shown to provide many health benefits due to their rich nutrient content, including polyphenols, proteins, amino acids, caffeine, vitamins, theaflavins, and minerals [142]. Generally, the quality of tea determines its economic value and impact on human health. However, tea quality is affected by light, warmth, water, and fertilizer during the growing process, as well as by environmental conditions during production and storage. In recent years, the HSI technique combined with various algorithms and models has been explored to assess tea quality and grade. Typically, Li et al. reported evaluating green tea quality using a combination of the HSI technique and olfactory visualization systems (Figure 10a) [143]. In their work, a support vector machine was proposed to fuse multisensory data, achieving an accuracy of 92%. Notably, tea quality has been further assessed by the HSI technique combined with additional models, including the TSPSO-ResNet-50 model with an accuracy of 92.31% [144], the 1D-ResNet18 model with an accuracy of 99.56% [145], and PLSDA with an accuracy of 100% [146] (Figure 10b–d). Additionally, the mold, including aspergillus and penicillium, which causes severe tea mildew during storage, has been rapidly detected by the HSI technique combined with CARS-GA-PSO-SVR algorithms, with an *R*_p_^2^ of 0.9577 and an RMSEP of 0.1140 lg(CFU/g) (Figure 10e) [147].

In addition to the quality of the tea leaves, the quality of the tea powder (i.e., matcha) has also been evaluated using the HSI technique. Ouyang et al. have reported rapid and accurate estimation of sensory attributes, such as appearance, infusion color, aroma, and taste, of matcha using the HSI technique (Figure 10f) [148]. In their work, an ANN model was constructed using characteristic wavelengths extracted by the CARS algorithm with an *R*_p_^2^ of 0.7774. Meanwhile, the same group further reported evaluating particle sizes in matcha using the HSI technique combined with CARS-ANN algorithms, achieving an accuracy greater than 80% [150]. Furthermore, they proposed BOSS-PLS models to simultaneously quantify chemical constituents in matcha, such as caffeine, tea polyphenols, free amino acids, and chlorophyll from HSI data [151]. In addition, Li et al. applied the HSI technique together with chemometrics and the iRF-SPA-PLS mixed algorithms to evaluate matcha’s sensory quality by analyzing its color physicochemical indicators (Figure 10g) [149]. Table 5 summarizes recent reports on the typical results of tea quality and grade assessment using the HSI technique.

### 3.2. Hyperspectral Imaging for Moisture Content Detection

**Figure 11:**
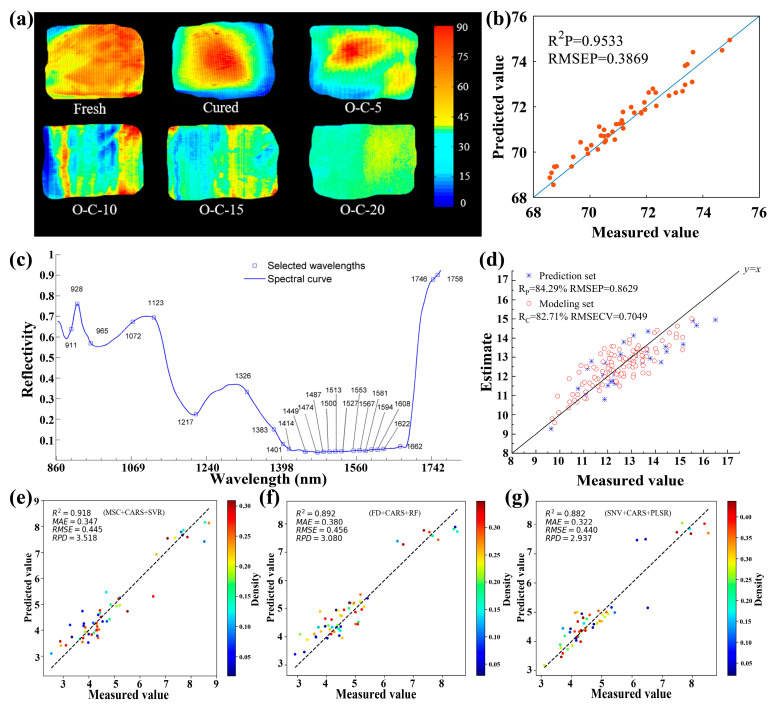
(**a**) Visualization of moisture content of fresh, cured, and oven-cooked (O-C) pork at varying cooking times: 5 h (O-C-5), 10 h (O-C-10), 15 h (O-C-15), and 20 h (O-C-20). Reproduced with permission from Ref. [157]. (**b**) Measured and predicted moisture content in frozen-thawed pork samples based on an improved decision fusion method. Reproduced with permission from Ref. [158]. (**c**) Characteristic wavelengths of rice moisture obtained from the SPA algorithm. Reproduced with permission from Ref. [27]. (**d**) Measured and predicted moisture content in the lettuce canopies by the MCUVE-CARS-PLS model [160]. (**e**–**g**) Measured and predicted moisture content in wheat leaves by the (**e**) MSC-CARS-SVR, (**f**) FD-CARS-RF, and (**g**) SNV-CARS-PLSR models [161].

Moisture plays a vital role in many foods, affecting their sensory qualities, processing techniques, food safety, and shelf life [156]. Typically, moisture content affects the juiciness, flavor, appearance, and texture of meat, which determines the quality of frozen and dry-cured meats. Additionally, the moisture content in grains such as rice and wheat affects their eating quality, storage time, and germination rate the following year. Nondestructive and rapid detection of moisture in foods is vital for ensuring food safety and supporting agricultural productivity. Recently, the HSI technique has been explored to detect the moisture content in various foods. Tian et al. have demonstrated rapid and accurate monitoring of moisture distribution in dry-cured pork using the HSI technique (Figure 11a) [157]. The CARS algorithm was used to extract characteristic wavelengths, based on which a PLSR model was built to predict the moisture content, with an *R*_p_^2^ of 0.926 and RMSEP of 0.121. Notably, Cheng et al. demonstrated the effective detection of moisture content in frozen-thawed pork using the HSI technique combined with an improved decision fusion, where the accuracy was increased to 0.9533 (Figure 11b) [158]. Sun et al. reported the effective detection of moisture content in rice using HSI technique combined with a BCC-LS-SVR-SPA mixed algorithm [27]. In their work, PCA and SPA algorithms were used to extract characteristic wavelengths (Figure 11c), based on which a BCC-LS-SVR model was constructed with accuracies of 0.980 and 0.985 on the prediction and calibration sets, respectively. In addition, the moisture content of both ice seeds and hulled rice has been further measured by the HSI technique [67,159]. Specifically, this was achieved by building the SAGA-SVR model for rice seeds and the MSLPP-ESMA-SVR model for hulled rice, respectively.

Additionally, the HSI techniques have been investigated for detecting water content in plants to monitor and evaluate their growth quality. Zhao et al. have reported the detection of water content in lettuce canopies based on the HSI technique combined with the Monte Carlo UVE and CARS algorithms to extract characteristic wavelengths and a PLS model to predict the water content with accuracies of 82.71% and 84.29%, for calibration and prediction sets, respectively (Figure 11d) [160]. Wu et al. reported the estimation of water content in wheat leaves using the HSI technique combined with ML methods [161]. In brief, CARS and Hilbert–Schmidt independence criterion lasso algorithms were used to extract characteristic wavelengths and eliminate redundant information, followed by building SVR, RF, and PLSR models to predict the water content with accuracies of 0.918, 0.892, and 0.882, respectively (Figure 11e–g). Additionally, the HSI technique has been employed to detect the moisture content of foods during their processing. Zhu et al. reported the determination of moisture content and total acid content in vinegar during solid-state fermentation using the HSI technique [162]. In their report, synergy interval PLS and GA-PLS algorithms were used to select optimum variables, and the gray-level co-occurrence matrix algorithm was employed to obtain optimum texture feature variables. After fusing these two features, a GA-PLS model was built to predict the water content and total acid content, with accuracies of 0.8565 and 0.8162, respectively. Tian et al. have reported the quantitative analysis and visualization of the moisture and anthocyanins content in purple sweet potatoes during their convective hot-air and microwave drying processes using the HSI technique combined with a PLSR model with predictive accuracy higher than 0.8 [84]. In addition, the same group further demonstrated the visualization of moisture content, reducing sugars, and chewiness in bread during oral processing using the HSI technique combined with the PLSR-SG model [163]. Table 6 presents the typical results for detecting moisture content in various foods using the HSI technique reported earlier.

### 3.3. Hyperspectral Imaging for Varieties and Origin Identification

**Figure 12:**
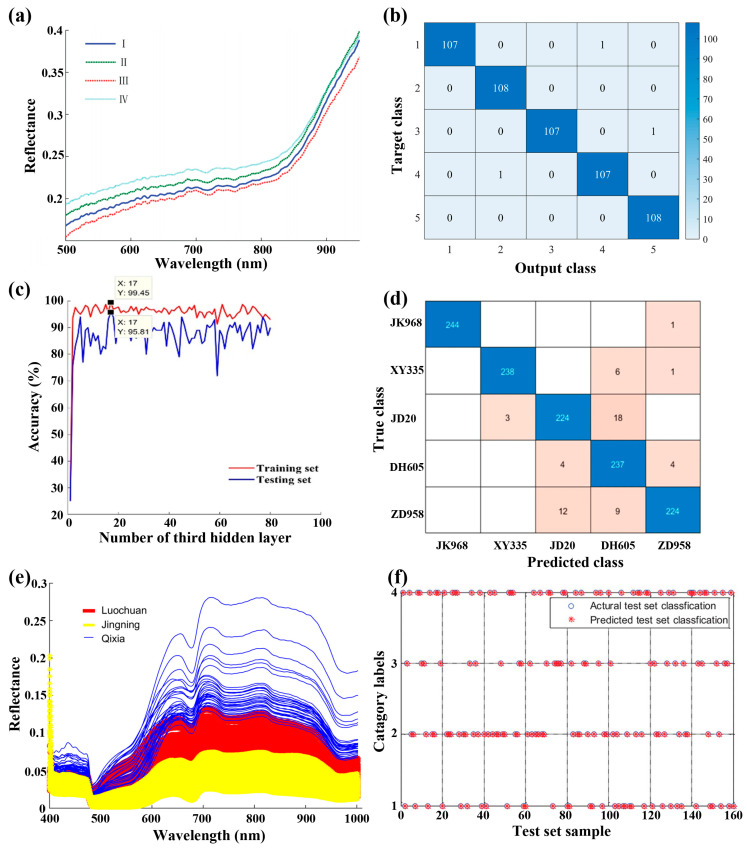
(**a**) Mean reflectance spectra of four rice origins, including Guangdong (I), Anhui (II), Heilongjiang (III), and Jiangsu (IV). Reproduced with permission from Ref. [168]. (**b**) Confusion matrix of the AFSA-SVM model for predicting rice seed varieties. Reproduced with permission from Ref. [169]. (**c**) Accuracies of the training and testing sets for the detection of maize seed varieties by the SSAE-CS-SVM model. Reproduced with permission from Ref. [172]. (**d**) Confusion matrix for the prediction of maize varieties with the CNN-LSTM model [173]. (**e**) Original spectral curves of the regions of interest of apples from different regions. Reproduced with permission from Ref. [174]. (**f**) Identification results of grape varieties based on the EEMD-DWT-CARS-SPA mixed algorithms. Reproduced with permission from Ref. [85].

The quality and economic value of various foods, such as grains and fruits, are closely related to their varieties and geographic origins, as climatic and geological conditions in the growth area affect both taste and nutritional composition [166,167]. Although many foods with different varieties may appear similar in terms of appearance, size, and color, their market prices can vary significantly. Nondestructive and rapid detection of the varieties and origins of grains and fruits is essential for maintaining market order and safeguarding consumers’ rights and interests. In recent years, HSI techniques with various algorithms and models have been used to rapidly and nondestructively assess the varieties and origins of grains and fruits. Typically, Sun et al. have shown the rapid identification of rice origins using the HSI technique (Figure 12a) [168]. In their work, spectral, morphological, and textural features were extracted from the obtained hyperspectral images, and an SVM model was built, achieving an accuracy of 91.67%. Very recently, the same group reported the detection of rice seed varieties. They employed a bootstrapping soft-shrinkage (BOSS) algorithm to extract the characteristic wavelengths from the spectral data, based on which the built SVM model showed an accuracy of 91.48% [169]. In another work, an SVM model built based on characteristic wavelengths extracted from the CARS algorithm has also shown high accuracy in predicting Lycium barbarum varieties [170]. Notably, an artificial fish swarm algorithm (AFSA) has been employed to optimize the SVM model, achieving an accuracy of 99.44% for predicting rice seed varieties [169] (Figure 12b). Additionally, by combining spectral and image features, the accuracy of PLS-DA and SVM prediction models can be further improved [171]. Furthermore, the HSI technique has been explored to assess the maize seed varieties. Fu et al. have reported the rapid detection of maize seed varieties based on the HSI technique and stacked sparse autoencoder combined with a cuckoo search (CS) optimized SVM (SSAE-CS-SVM) models, with an accuracy of 99.45% and 95.81% for predicting the training and testing sets, respectively (Figure 12c) [172]. Recently, the DL technique has also been explored to decode the hyperspectral image data to identify maize seed varieties. Typically, Zhu et al. have reported a CNN–Long Short-Term Memory (LSTM) model for identifying maize seed varieties, with an accuracy of 95.27% (Figure 12d) [173].

In addition to grain varieties, fruit varieties have also been identified using the HSI technique combined with various algorithms and models. For example, Tian et al. have reported the rapid identification of apple varieties using the HSI technique (Figure 12e) [174]. In their study, VISSA-SR mixed algorithms were employed to extract characteristic wavelengths; based on them, SVM was employed to build a prediction model, achieving accuracies of 100% and 97.14% for calibration and prediction sets, respectively. Recently, Xu et al. demonstrated the effective identification of grape varieties using the HSI technique combined with the EEMD-DWT-CARS-SPA mixed algorithms, achieving an accuracy of 100% (Figure 12f) [85]. Notably, Wang et al. reported that similar algorithms, specifically the CARS-IRIV-SSA-SVM mixed algorithms, can identify red jujube varieties, achieving accuracies of 100% and 96.68% for the training set and testing set, respectively [133]. Table 7 presents the typical results of the identification of grain and fruit varieties using the HSI technique combined with various algorithms and models reported in recent years.

Furthermore, the HSI technique has also been explored to identify tea varieties. Typically, Sun et al. reported the nondestructive and rapid identification of green tea varieties [175]. In their report, the VISSA algorithm was used to extract characteristic wavelengths, and FA-SVM mixed algorithms were employed to build prediction models, achieving an accuracy of 100% and 96% for prediction of calibration and prediction sets, respectively. Additional algorithms, including BOSS-light gradient boosting machine (LightBGM) [176] and CARS-ABC-SVM [177] mixed algorithms, have been explored for the prediction of oolong tea varieties, with accuracies higher than 97%. Typical achievements in identifying tea varieties reported in recent years are summarized in Table 7.

**Table 7:** Typical results of recent grain, fruit, and tea varieties identification using the HSI technique.

Objective	Accuracy for Training Set (*R*^2^)	Accuracy for Test Set (*R*^2^)	Ref.
Green tea variety	100%	96%	[175]
Red jujube variety	100%	96.68%	[178]
Apple origin	100%	97.14%	[174]
Grape variety	100%	99.3125%	[85]
Oolong tea variety	100%	97.33%	[176]
Tea variety	100%	100%	[177]
Maize seed variety	100%	95.27%	[173]
Black bean variety	-	98.33%	[171]
Lycium barbarum variety	100%	85%	[170]
Rice seed variety	100%	99.44%	[169]
Pu’er ripe tea variety	100%	96.50%	[179]

**Note.** The reported performance metrics are study-specific and should be interpreted with caution.

### 3.4. Hyperspectral Imaging for Additive and Adulteration Detection

**Figure 13:**
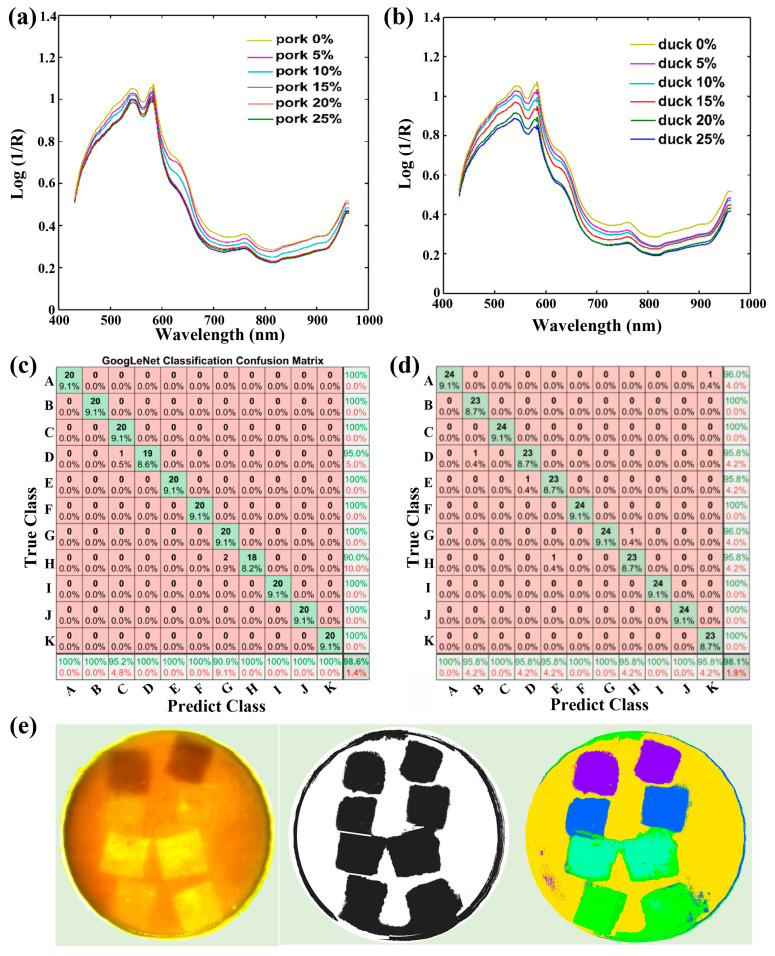
(**a**,**b**) Average spectrum of steak samples with varying levels of (**a**) pork and (**b**) duck adulterations [181]. Confusion matrix of (**c**) GoogLeNet network for starch detection in minced chicken meat (reproduced with permission from Ref. [182]) and (**d**) VGG16-SVM mixed algorithms for soybean protein identification in minced chicken meat (reproduced with permission from Ref. [86]). (**e**) Visualization of analogous density foreign materials, including polyethylene terephthalate, polyvinyl chloride, polylactic acid, and polypropylene, inside semi-finished soy protein meat (reproduced with permission from Ref. [183]).

As the population grows, the market’s demand for food increases. To maximize profits, some merchants may deliberately add additives or adulterate foods with foreign materials and toxic substances. This poses health risks to consumers, such as allergic reactions and illnesses. Nondestructive, rapid, and reliable detection of additives and adulteration is necessary. Recently, the HSI technique combined with various algorithms and models has been explored to nondestructively and rapidly detect various additives and adulteration in various meats. In 2013, Kamruzzaman et al. reported the effective detection of adulterations, such as pork, heart, kidney, and lung, in minced lamb meat using the HSI technique [180]. Recently, Liu et al. reported the quantitative detection of adulteration, including minced pork and duck, in the restructured steak using the HSI technique (Figure 13a,b) [181]. In their report, iRF-CARS mixed algorithms were used to extract characteristic wavelengths, based on which a PLS prediction model was built, achieving accuracies of 98.49% and 98.21% for predicting pork and duck, respectively. Yang et al. have reported the effective detection of starch additives in minced chicken meat using the HSI technique combined with the GoogLeNet network (Figure 13c) [182]. The prediction model exhibited an accuracy of 98.6%, which is higher than that of the SVM and 2D-CNN models, i.e., 95.9% and 89.55, respectively. Recently, Sun et al. reported the nondestructive identification of soybean protein, which is a typical plant-based artificial meat, using the HSI technique combined with VGG16-SVM mixed algorithms, achieving an accuracy of 98.1% (Figure 13d) [86]. In another work, analogous density foreign materials, including polyethylene terephthalate, polylactic acid, polypropylene, and polyvinyl chloride, in soybean protein have been effectively detected using the HSI technique combined with SVM-PCA-MSC-SPA mixed algorithms (Figure 13e) [183]. Notably, the MSC method was employed to preprocess the extracted spectra to eliminate nonlinear baseline drift, which increased the accuracy of the prediction model to 95%.

Additionally, the HSI technique has been explored to detect additives and adulterations in dried foods. Typically, Cai et al. have shown effective detection of cow milk powder adulterations in goat milk powder [184]. The LassoNet algorithm was employed to extract characteristic wavelengths, based on which a BWO-SVM model was constructed with an accuracy of 94.55%. Tang et al. have reported the identification of fumigated and dyed lyceum barbarum using the HSI technique, in which the latter is generally involved with sulfur and Sudan red, which severely harm human health [185]. In their report, the CARS algorithm was used to extract the characteristic wavelengths, and then a SVM model was built and optimized by the slime mold algorithm, achieving accuracies of 98.2 and 96.7% for the training and testing sets. Notably, the prediction accuracy has been improved to 100% for both training and testing sets by using the GA algorithm to optimize the SVM model [186]. In addition, the HSI technique has been employed to identify homochromatic foreign materials, such as transparent plastic, homochromatic plastic and paper from packaging material, and homochromatic rubber from the rubber bands that bind the tobacco leaves, in cut tobacco leaves, where a PCA algorithm was used to extract characteristic wavelengths to build a back-propagation ANN model, achieving an accuracy of 100% [187]. Additionally, Zhang et al. have shown effective detection of saccharin jujube adulterations in winter jujube samples, with an accuracy of 91.67%, using the HSI technique combined with VISSA-GWO-SVM mixed algorithms [188]. Table 8 shows typical results for the identification of additives and adulterants in various foods using the HSI technique with various algorithms and models.

### 3.5. Hyperspectral Imaging for Heavy Metal and Pesticide Residue Detection

**Figure 14:**
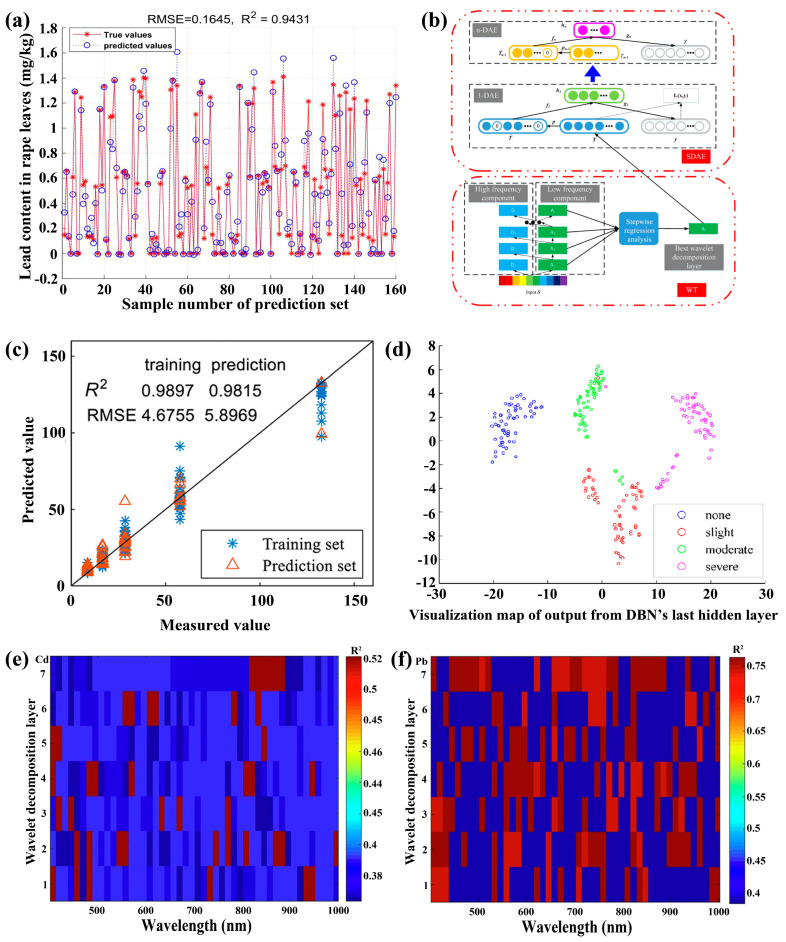
(**a**) Prediction of the lead content in rape leaves based on a modified RF-HHO-SVR model. Reproduced with permission from Ref. [192]. (**b**) Typical flow chart of the wavelet transform and stacked denoising autoencoder algorithm. Reproduced with permission from Ref. [28]. (**c**) Measured and predicted cadmium content in rape leaves by the stacking random forest model (i.e., using SVR, extreme learning machine, decision tree, and random forest as basic learners and using random forest as a meta learner for stacking). Reproduced with permission from Ref. [193]. (**d**) Visualization maps of lettuce under different Pb stress gradients (i.e., none, slight, moderate, and severe). Reproduced with permission from Ref. [196]. (**e**,**f**) Coefficient of determination for predicting (**e**) Cd and (**f**) Pb content in lettuce based on WT and SCAE algorithms. Reproduced with permission from Ref. [197].

In recent years, industrialization and modern agriculture have increased the difficulty of avoiding baseline soil and water pollution. Heavy metals have accumulated in farmland over time from industrial waste, wastewater irrigation, and legacy sources such as leaded gasoline. Furthermore, pesticides have been widely applied during planting and storage to enhance yield and appearance. Over time, some residues may enter the food chain via degradation, illegal application, or environmental persistence. As a result, nondestructive and rapid detection of heavy metals and pesticides is vital for human health. Recently, the HSI technique has been widely studied for the nondestructive and rapid detection of heavy metals in crops and vegetables. Typically, Cao et al. have reported the effective detection of lead content in oilseed rape leaves using the HSI technique [192]. A modified RF was studied to extract characteristic wavelengths, followed by constructing a Harris Hawks Optimizer (HHO)-SVM model with an *R*_p_^2^ of 0.9431 and RMSEP of 0.1645 mg/kg (Figure 14a). In another report, the DL method, involving wavelet transform and stacked denoising autoencoder (SDAE, Figure 14b), was further explored to extract the deep features of lead in oilseed rape. Based on this, an SVR model was built with an *R*_p_^2^ of 0.9388 and RMSEP of 0.0199 mg/kg [28]. In addition to lead, the HSI technique has been used to effectively detect cadmium and copper content in oilseed rape. Cheng et al. have reported detecting cadmium content using the HSI technique combined with ensemble learning methods [193]. In their report, two-layer estimation models were proposed using SVR, extreme learning machine, decision tree, and random forest as base learners, with random forest serving as a meta learner, achieving a high accuracy with an R_p_^2^ of 0.9815 and an RMSEP of 5.8969 mg/kg (Figure 14c). Recently, the cadmium content in oilseed rape leaves across different silicon environments has been effectively detected by using the HSI technique combined with a transfer-stack denoising autoencoder algorithm [194]. Additionally, Peng et al. have reported the classification of copper stress levels in oilseed rape using the HSI technique combined with deep residual networks, achieving an accuracy exceeding 98% [195].

The HSI technique has been studied for the effective detection of various heavy metals in vegetables. Typically, Sun et al. have reported an evaluation of lead pollution levels in lettuce leaves using the HSI technique. In their report, a deep belief network (DBN) was built with accuracies of 100% and 96.67% for training and testing sets, respectively (Figure 14d) [196]. Zhou et al. have presented visualizing cadmium content in lettuce leaves using the HSI technique combined with a wavelet SVM regression model, with an R_p_^2^ of 0.8843 and an RMSEP of 0.1292 mg/kg [169]. In another report, the CNN method was employed to extract characteristic wavelengths, on which an LSSVR model was built for predicting cadmium content in lettuce leaves, with an R_p_^2^ of 0.9044 and an RMSEP of 0.0255 mg/kg [88]. Notably, the HSI technique has been further explored to accurately detect both lead and cadmium content in lettuce leaves [197]. Specifically, a DL method combining WT and SCAE algorithms was used to extract deep features for lead and cadmium detection, on which an SVR model was built, achieving R_p_^2^ values of 0.9319 and 0.9418, and RMSEP values of 0.04988 mg/kg and 0.04123 mg/kg for cadmium and lead, respectively (Figure 14e,f).

**Figure 15:**
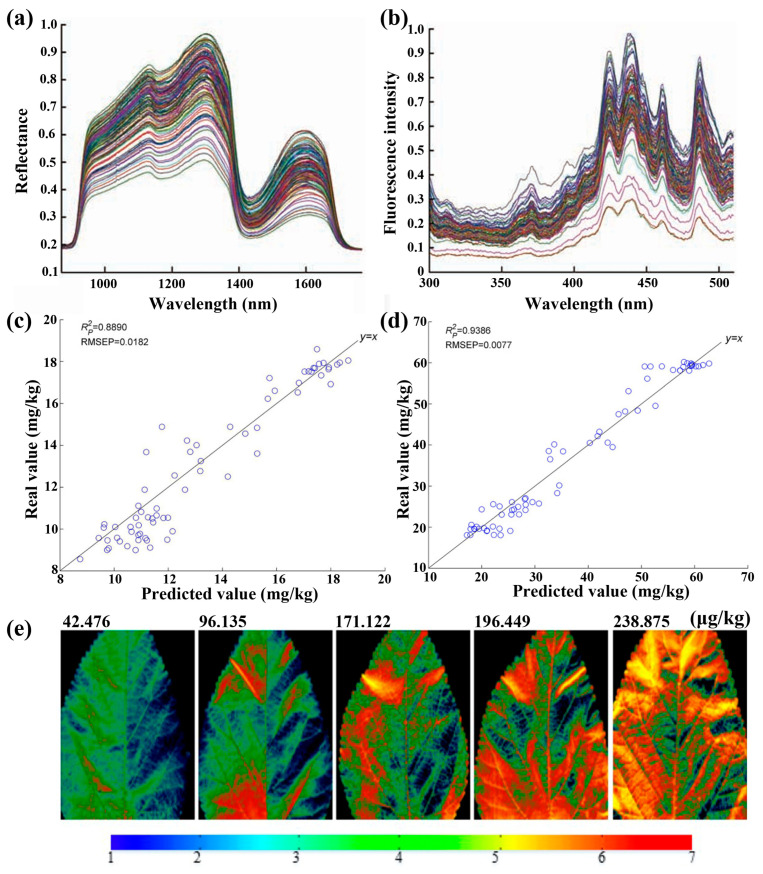
(**a**,**b**) Hyperspectral curves of lettuce leaves in (**a**) near-infrared and (**b**) visible spectral range [171]. (**c**,**d**) Predicted and real values of (**c**) fenvalerate and (**d**) dimethoate in lettuce leaves by SPA-LSSVR models. Reproduced with permission from Ref. [198]. (**e**) Distribution map of pesticide residues in mulberry leaves containing different chlorpyrifos residues as determined by the SPA-MLR model. Reproduced with permission from Ref. [199].

In addition, the HSI technique has been explored for the effective and rapid detection of pesticide residues in various vegetables. Sun et al. have demonstrated the detection of dimethoate concentrations in lettuces using the visible and near-infrared HSI technique coupled with chlorophyll fluorescence spectra (Figure 15a,b) [171]. In brief, the wavelet transform (WT) and the MD-MCCV algorithm were developed to extract characteristic wavelengths, based on which an SVR model was constructed for predicting dimethoate concentrations with an R_p_^2^ of 0.987 and an RMSEP of 0.005. Notably, mixed pesticides in lettuce, such as fenvalerate and dimethoate, have been accurately detected by the HSI technique [198]. In their report, two different kinds of characteristic wavelengths were extracted from the CARS and random forest-recursive feature elimination algorithm, respectively. Then, SPA-LSSVR models were constructed based on these two types of characteristic wavelengths to separately predict fenvalerate and dimethoate, achieving high accuracies with R_p_^2^ values of 0.8890 and 0.9386 and RMSEP values of 0.0182 and 0.0077 for predicting fenvalerate and dimethoate, respectively (Figure 15c,d). Furthermore, chlorpyrifos EC, a common organophosphorus pesticide, has been effectively detected by the HSI technique. Jiang et al. have reported the visualization of chlorpyrifos EC content in mulberry using the HSI technique combined with the SPA-MLR mixed algorithms (Figure 15e) [199]. In brief, the SPA algorithm was used to extract the characteristic wavelengths, based on which an MLR model was constructed to accurately detect the chlorpyrifos EC content with an R_p_^2^ of 0.859 and an RMSEP of 38.789. Table 9 presents typical results for the nondestructive and rapid detection of various heavy metals and pesticide residues in crops and vegetables using the HSI technique reported recently. Generally, HSI has a higher detection limit, i.e., lower analytical sensitivity, than mass spectrometry or chromatography-based confirmatory methods, but it offers important advantages in terms of rapid, nondestructive, and spatially resolved screening, making it valuable in food and agricultural applications.

### 3.6. Others

The HSI technique has been further developed to effectively and rapidly evaluate seed viability and plant growth, both of which are vital for agricultural production. Recently, Sun et al. presented the rapid and nondestructive detection of watermelon seed viability using the HSI technique combined with the ML algorithm [203]. In brief, a PCA algorithm was utilized to extract the characteristic wavelengths, based on which an SVM model was built with an accuracy of 100% and 92.33% for the prediction and test sets, respectively. Notably, both accuracies increased to 100% after optimizing the SVM model using an artificial bee colony (ABC) algorithm. In assessing the plant growth status, the total nitrogen content was detected by the HSI technique, considering that nitrogen is an important component of protein, nucleic acid, and chlorophyll. Zhu et al. reported the effective detection of total nitrogen and soluble sugars in tomato leaves using the HSI technique to assess tomato nutrient stress [204]. In their work, the PCA algorithm was used to reduce the spectral dimension and extract the characteristic wavelengths. Then, linear (MLR and PLS) and nonlinear (SVM and BPANN) prediction models were constructed with accuracies as high as 90%. Very recently, Zhang et al. reported monitoring soybean growth by detecting nitrogen content in the soybean canopy using the HSI technique [126]. Additionally, hyperspectral imaging data collected from unmanned aerial vehicles were used to create a spatial distribution map of soybean nitrogen content at the flowering and seed-filling stages.

The HSI technique has also been explored for evaluating plant diseases. Tao et al. reported the effective classification of the hazard level of brown planthopper damage in rice using the HSI technique [205]. In brief, SG smoothing and PCA algorithms were used to extract characteristic wavelengths, and then a broad learning system (BLS) algorithm was employed to develop the prediction model with an accuracy of 99.08% and a precision of 99.31%. In addition, tea white star disease and anthrax, which are similar in their imaging features, have been accurately identified using the HSI technique combined with a spectrum extraction method based on the region-of-interest spots [206]. Very recently, Zhang et al. demonstrated the detection of tomato leaf mildew using HSI and THz time-domain spectroscopy, with the characteristic wavelengths extracted from the GA and PCA algorithms, respectively [207]. Notably, a fusion diagnosis and health evaluation model for tomato leaf mildew has been developed using hyperspectral fusion with THz, achieving an accuracy as high as 97.12%. Collectively, the HSI method has become integrated into all aspects of food quality and agricultural production in recent years.

## 4. Challenges and Outlook

In summary, HSI technology has demonstrated significant promise in food quality and safety detection in recent years, benefiting from its ability to provide unified spectral data. Remarkable progress has been achieved in various areas, including food quality, pesticide residue analysis, adulteration and additive detection, and freshness assessment. However, HSI technology still faces several significant challenges that need attention.

Initially, there are challenges in data and hardware systems. This is related to the data cube structure of HSI data, which combines detailed spectral data with two-dimensional spatial information (Figure 4). This results in exponential growth in data volume, creating large data cubes that require storage and real-time processing resources. Furthermore, the HSI systems face an inherent challenge in balancing high spectral and spatial resolutions with a high signal-to-noise (SN) ratio, necessitating complex design trade-offs [208,209]. This certainly results in high hardware costs and large sizes, while requiring strict standards for platform stability and calibration accuracy.

Secondly, there are challenges in information mining and algorithms. The high dimensionality of HSI data leads to the curse of data dimensionality. Traditional algorithms tend to overfit when handling hundreds of highly correlated spectral bands, and phenomena such as “same material different spectra” or “different materials same spectra” are common, greatly limiting the accuracy of object classification and identification [210]. Although deep learning models have strong fitting abilities, they require a large number of training samples. Generally, techniques such as transfer learning and domain adaptation can be employed to leverage abundant source-domain data to model the target domain [211]. In addition, self-supervised and contrastive learning paradigms can be further studied to extract spectral features from unlabeled data, thereby decreasing reliance on labeled samples [212,213].

Finally, there are challenges in application implementation and industrialization. HSI technology faces a gap between laboratory research and deployment in complex scenarios. In outdoor settings, factors such as variable lighting, atmospheric disturbances, and terrain variations can significantly influence spectral stability, often leading to model failures in practical applications [214,215]. In addition, high system expenses, slow data collection, and complex operation procedures limit the widespread adoption of HSI in large-scale civilian sectors, such as food quality and safety detection. Developing inexpensive, miniaturized, real-time online detection systems and creating standardized data-sharing and model-transfer frameworks remain significant challenges at the current stage. These obstacles continue to limit the broader practical adoption of this technology.

## 5. Conclusions

This review has systematically summarized recent progress in the HSI technique for the nondestructive and effective detection of food quality and safety. By combining imaging with spectroscopy, HSI simultaneously provides spatial and spectral data, enabling real-time and highly sensitive assessment of both internal and external qualities of vegetables, fruits, meat, grains, and teas, including chemical composition, moisture content, ripeness, variety and origin, adulterants, additives, heavy metals, and pesticide residues. It showed that the use of HSI combined with machine learning and deep learning algorithms significantly improves the extraction of characteristic wavelengths and the accuracy of predictive models, with most laboratory studies achieving over 90% prediction accuracy. Meanwhile, challenges and perspectives regarding the high dimensionality of HSI data, the trade-off between resolution and signal-to-noise ratio, hardware costs, and the gap between laboratory research and applications under variable environmental conditions are proposed. It has been suggested that future research can focus on developing inexpensive, miniaturized, and real-time online HSI systems, advancing self-supervised, transfer, and contrastive learning paradigms to reduce dependence on large-scale labeled datasets, and establishing standardized frameworks for data sharing and model transfer. Collectively, the HSI technique is considered an essential tool for detecting and monitoring food quality and safety in agricultural production.

## Figures and Tables

**Table 1:** Typical characteristic wavelengths and key roles of typical pigment compounds. Note that these characteristic wavelengths may vary with pH and temperature.

Pigment Compounds	Characteristic Wavelengths	Roles
Chlorophyll	~430 and ~660 nm in absorption spectra; ~550 nm in transmission and reflection spectra.	Assessment of the freshness and maturity of most vegetables and fruits.
Carotenoids	400–500 nm with peaks of ~450 and 480 nm in absorption spectra; 500–700 nm in transmission and reflection spectra.	Assessment of the maturity and nutrient content of tomatoes, corn, and citrus fruits.
Anthocyanins	~520–550 nm and ~600 nm in the absorption spectra.	Detection of the quality of berries, such as grapes and kiwifruit, as well as certain vegetables.
Myoglobin	~416, ~542–549, and ~575–587 nm (oxymyoglobin); ~430–435, ~555–560, and ~760 nm (deoxymyoglobin); ~408–423, ~500–505, ~540–580, 630, and 760 nm (metmyoglobin). All of them are included in the absorption spectra.	Assessment of the freshness and quality of meat.
Hemoglobin	~414–415, ~540–542, and ~577–580 nm (oxyhemoglobin); ~430–432, ~555–560, and 760 nm (deoxyhemoglobin); ~406–420, ~500, ~540, ~578, and ~630 nm (methemoglobin); ~419–421, ~538–540, ~569–572 nm (carboxyhemoglobin). All of them are included in the absorption spectra.	Evaluation of meat freshness, quality, and blood oxygen levels.

**Table 2:** Typical methods (such as machine learning and deep learning) for HSI data preprocessing, characteristic wavelength extraction, and predictive model construction shown in recent years.

Objective	Data Preprocessing	Characteristic Wavelength Extraction	Predictive Model Construction	Ref.
tomato maturity and quality	SNV	CARS	SVC, SVR, PLSR	[69]
grape quality	SNV, FD	UVE, CARS	DBO, SABO, WOA, ELM	[70]
soluble solid content in apples	SG, SNV, DT	SPA, CARS	GWO, SVR	[71]
S-ovalbumin content in egg	SNV	CARS	PLSR, LSSVM	[72]
egg freshness	MSC, SNV, MC, MA, DFA, SG, SG FD, SG, SD, autoscales, normalization,	CARS, PCA, SPA	SVM, KNN, RF, NB, DAC, LDirA	[73]
egg freshness	SG	SPA, BOSS	HHO, SVR	[74]
egg quality	SNV	SPA, IRIV	SVM, XGBoost	[75]
salted duck egg quality	SG, SNV, MSC	CARS, UVE	PLS	[76]
microbial colony counting	SNV	GA, PCA	KNN	[77]
edible bird’s nest quality	SNV	GA-iPLS, GA-PLS	GA-iPLS, GA-PLS	[78]
panax notoginseng powder grades	SG, MSC	CARS, PCA	LSSVM, MPA, LSSVM	[79]
prepared dishes quality	DFA, SG, SNV	PCA	FTC, SVM, KNN	[80]
Yunnan coffee bean quality	DT, SNV, SG	PCA, WT	ECA, MobileNetV3	[81]
chemical compositions in shrimp flesh deterioration	SNV, MSC, FD, SD, SG	CARS, IRIV, VCPA, IRIV	PLS, LSTM	[82]
frozen-thawed pork quality	MSC, VMD, OSC, SG-Der	MI, VIF	PLSR	[83]
moisture and anthocyanins content in purple sweet potato	-	CARS	PLSR	[84]
grape variety	EEMD, DWT	CARS, SPA	SVM	[85]
soybean protein in minced chicken meat	SG, SNV, CWT	VGG16	SVM, CNN	[86]
heavy metal lead in eggs	SG, SNV, FD	VMD, SAE	LSSVR	[87]
heavy metal cadmium in lettuce	SG, FD	CARS, IRIV, VISSA	LSSVR	[88]

**Note:** Standard normal variable (SNV), competitive adaptive reweighted sampling (CARS), support vector classifier (SVC), support vector regression (SVR), partial least squares regression (PLSR), first-derivative (FD), second derivative (SD), uninformative variable elimination (UVE), dung beetle optimization (DBO), whale optimization algorithm (WOA), subtraction-average-based optimization (SABO), detrending (DT), extreme learning machine (ELM), Savitzky–Golay smoothing (SG), successive projection algorithm (SPA), gray wolf optimization (GWO), least squares support vector machine (LSSVM), multiplicative scatter correction (MSC), mean centering (MC), moving average method (MA), principal component analysis (PCA), orthogonal signal correction (OSC), Savitzky–Golay derivative (SG-Der), variational mode decomposition (VMD), detrend fluctuation analysis (DFA), bootstrapping soft shrinkage (BOSS), k-nearest neighbor (KNN), random forest (RF), Naïve Bayes (NB), discriminant analysis classifier (DAC), latent Dirichlet allocation (LDirA), iteratively retains informative variable (IRIV), Harris hawks optimization (HHO), extreme gradient boosting (XGBoost), genetic algorithm (GA), marine predators algorithm (MPA), fine tree classifier (FTC), wavelet transform (WT), variable combination population analysis (VCPA), long short-term memory (LSTM), efficient channel attention (ECA), mutual information (MI), discrete wavelet transform (DWT), variance inflation factor (VIF), continuous wavelet transform (CWT), stacked autoencoder (SAE), ensemble empirical mode decomposition (EEMD), least squares support vector regression (LSSVR), and variable iterative space shrinkage approach (VISSA).

**Table 3:** Typical results of recent quality assessments of fruits and vegetables using the HSI technique.

Objective	Accuracy for Training Set		Accuracy for Test Set		Ref.
	*R* ^2^	RMSEC	*R* ^2^	RMSEP	
Lycopene in tomatoes	0.9826	0.0079 mg/kg	0.9652	0.0166 mg/kg	[69]
Selenium in lettuces	0.9542	0.0361 mg/kg	0.8975	0.0487 mg/kg	[99]
Anthocyanins in lettuces	0.8623	0.0098 mg/g	0.8617	0.0095 mg/g	[70]
Solanine in potatoes	-	-	0.9143	0.0296	[97]
External defects in potatoes	75.5%	-	93.1%	-	[106]
Defects of tomatoes	-	-	97.69%	-	[107]
Lycopene in cherry tomatoes	0.95	8.75 mg/kg	0.93	10.33 mg/kg	[108]
Total soluble solid in grape	0.96	0.0045	0.93	0.006	[92]
Chilling injury in kiwifruit	100%	-	99.17%	-	[100]
Titratable in grape	0.9418	0.0962 g/L	0.9216	0.1091 g/L	[93]
Total soluble solid in apple	0.8713	0.5881 °Brix	0.8526	0.6262 °Brix	[109]
Total soluble solid in pears	0.8690	0.7092%	0.8731	0.7976%	[110]
Sucrose in melon	-	-	0.958	8.776	[111]

The reported performance metrics are study-specific and should be interpreted with caution.

**Table 5:** Typical results of recent tea quality assessment using the HSI technique.

Objective	Accuracy for Training Set		Accuracy for Test Set		Ref.
	*R* ^2^	RMSEC	*R* ^2^	RMSEP	
Quality of matcha	0.8433	2.05	0.7774	2.56	[148]
Tea polyphenols in matcha	0.6937	1.23%	0.7098	1.15%	[151]
Caffeine in matcha	0.8268	0.27%	0.8077	0.22%	[151]
Free amino acids in matcha	0.8114	0.38%	0.7942	0.37%	[151]
Mold in green tea	0.9605	0.104 lg(CFU/g)	0.9577	0.114 lg(CFU/g)	[147]
ECG in green tea	0.9673	1.2252	0.8746	2.2980	[146]
Tea polyphenols in green tea	0.89	-	0.75	-	[145]
Crude fiber in green tea	0.87	-	0.75	-	[145]
Grades of green tea	0.92	-	0.92	-	[143]
Quality of Maofeng tea	1.00	-	0.9231	-	[144]
Quality of fresh tea	0.8652	0.5304	0.8814	0.4597	[152]
Synthetic pigments in black tea	>0.95	<0.020	>0.95	<0.025	[153]
Classification of tea	-	-	97.41%	0.16%	[154]
Quality of black tea	99.78%	-	99.57%	-	[155]

**Note:** ECG (epicatechin gallate) is a key taste compound in tea. The reported performance metrics are study-specific and should be interpreted with caution.

**Table 6:** Typical results of recent moisture content detection in various foods using the HSI technique.

Objective	Accuracy for Training Set		Accuracy for Test Set		Ref.
	*R* ^2^	RMSEC	*R* ^2^	RMSEP	
Water content in lettuce	82.71%	0.7049	84.29%	0.8629	[160]
Water in wheat leaf	0.713	0.793	0.918	0.445	[161]
Moisture content in SSF	0.84	5.12 mg/g	0.82	5.36 mg/g	[162]
Moisture in potatoes	0.928	0.058 mg/g	0.834	0.109 mg/g	[84]
Moisture in bread	0.8926	1.8751	0.8898	2.0526	[163]
Moisture in dried pork	0.967	0.127	0.937	0.824	[157]
Moisture in frozen pork	-	-	0.9533	0.3869	[158]
Moisture in rice	0.985	0.591%	0.980	0.967%	[27]
Moisture in rice with husk	0.9828	0.7552%	0.9755	0.8597%	[67]
Moisture in rice seed	0.9536	0.0204	0.9318	0.0264	[159]
Moisture in tea leaves	0.963	0.023	0.941	0.031	[164]
Moisture in oilseed rape leaves	0.9717	0.0049	0.9555	0.0065	[165]

**Note:** SSF means solid-state fermentation processes. The reported performance metrics are study-specific and should be interpreted with caution.

**Table 8:** Typical results of recent identification of additives and adulterants in various foods using the HSI technique.

Objective	Accuracy for Training Set (*R*^2^)	Accuracy for Test Set (*R*^2^)	Ref.
Fraud in Mānuka honey	-	100%	[189]
Additives in SPM	96.67%	95%	[183]
SPM in chicken meat	99.1%	98.1%	[86]
Adulteration in steak	0.987	0.9835	[181]
Starch in minced chicken	99.4%	98.6%	[182]
Saccharin jujube in jujube	99.44%	91.67%	[188]
Additives in tobacco	-	100%	[187]
Adulteration in wolfberry	98.2%	96.7%	[185]
Adulteration in goat milk	95.76%	94.55%	[184]
Talcum powder in flour	0.98	0.98	[190]
Benzoyl peroxide in flour	-	0.9902	[191]

**Note:** SPM (soy protein meat) is artificial meat. The reported performance metrics are study-specific and should be interpreted with caution.

**Table 9:** Typical results for the detection of various heavy metals and pesticide residues in crops and vegetables using the HSI technique reported recently.

Objective	Accuracy for Training Set		Accuracy for Test Set		Ref.
	*R* ^2^	RMSEC	*R* ^2^	RMSEP	
Cadmium in lettuce leaves	0.9589	0.0178 mg/kg	0.9044	0.0255 mg/kg	[88]
Lead pollution in lettuce leaves	100%	-	96.67%	-	[196]
Copper pollution in oilseed rape	-	-	-	98.15%	[195]
Cadmium in oilseed rape leaves	0.9878	0.00532 mg/kg	0.9273	0.01465 mg/kg	[194]
Lead in oilseed rape leaves	0.9768	0.0084 mg/kg	0.9388	0.0199 mg/kg	[28]
Copper pollution in rice	-	-	0.74	2.10	[200]
Cadmium in rice	0.9998	5.93 mg/kg	0.9958	29.58 mg/kg	[201]
Dimethoate residue in lettuce	0.997	0.008	0.987	0.005	[171]
Chlorpyrifos EC in mulberry	0.889	34.427	0.859	38.789	[199]
Fenvalerate in tobacco	-	-	0.918	-	[202]

**Note.** The reported performance metrics are study-specific and should be interpreted with caution.

## Data Availability

No new data were created or analyzed in this study.
